# In vitro antiplasmodial activity, pharmacokinetic profiles and interference in isoprenoid pathway of 2-aniline-3-hydroxy-1.4-naphthoquinone derivatives

**DOI:** 10.1186/s12936-018-2615-8

**Published:** 2018-12-19

**Authors:** Valeska S. de Sena Pereira, Flávio da Silva Emery, Lis Lobo, Fátima Nogueira, Jonas I. N. Oliveira, Umberto L. Fulco, Eudenilson L. Albuquerque, Alejandro M. Katzin, Valter F. de Andrade-Neto

**Affiliations:** 1 Laboratório de Biologia da Malária e Toxoplasmose - LABMAT, Departmento de Microbiologia e Parasitologia, Universidade Federal do Rio Grande do Norte, Natal, RN Brazil; 2 Departmento de Ciências Farmacêuticas, Faculdade de Ciências Farmacêuticas de Ribeirão Preto, Universidade de São Paulo, Ribeirão Preto, SP Brazil; 3 Global Health and Tropical Medicine-GHTM, Unidade de Ensino e Investigação de Parasitologia Médica, Instituto de Higiene e Medicina Tropical, IHMT, Universidade Nova de Lisboa - UNL, Lisbon, Portugal; 4 Departamento de Biofísica e Farmacologia, Universidade Federal do Rio Grande do Norte, Natal, RN Brazil; 5 Departmento de Parasitologia, Centro de Ciências Biomédicas, Universidade de São Paulo, São Paulo, SP Brazil; 6 Post-graduate Program in Biochemistry, Universidade Federal do Rio Grande do Norte, Natal, RN Brazil

**Keywords:** *Plasmodium falciparum*, Antimalarial drugs, Hydroxy-naphthoquinones, ADME predictions, Isoprenoid pathway

## Abstract

**Background:**

*Plasmodium falciparum* has shown multidrug resistance, leading to the necessity for the development of new drugs with novel targets, such as the synthesis of isoprenic precursors, which are excellent targets because the pathway is different in several steps when compared with the human host. Naphthoquinone derivatives have been described as potentially promising for the development of anti-malarial leader molecules. In view of that, the focus in this work is twofold: first, evaluate the in vitro naphthoquinone antiplasmodial activity and cytotoxicity; secondly, investigate one possible action mechanism of two derivatives of hydroxy-naphthoquinones.

**Results:**

The two hydroxy-naphthoquinones derivatives have been tested against *P. falciparum* in vitro, using strains of parasites chloroquine-sensitive (3D7) and chloroquine-resistant (Dd2), causing 50% inhibition of parasite growth with concentrations that varied from 7 to 44.5 μM. The cell viability in vitro against RAW Cell Line displayed IC_50_ = 483.5 and 714.9 μM, whereas, in primary culture tests using murine macrophages, IC_50_ were 315.8 and 532.6 μM for the two selected compounds, causing no haemolysis at the doses tested. The in vivo acute toxicity assays exhibited a significant safety margin indicated by a lack of systemic and behavioural toxicity up to 300 mg/kg. It is suggested that this drug seems to inhibit the biosynthesis of isoprenic compounds, particularly the menaquinone and tocopherol.

**Conclusions:**

These derivatives have a high potential for the development of new anti-malarial drugs since they showed low toxicity associated to a satisfactory antiplasmodial activity and possible inhibition of a metabolic pathway distinct from the pathways found in the mammalian host.

## Background

Malaria still remains a major parasitic disease in the tropical and subtropical regions of the world due to its economic impact and high morbidity. In 2015, according to the latest estimates from the World Health Organization (WHO), there were 214 million new cases resulting in 438,000 deaths, mostly children under 5 years of age [1]. Although several anti-malarials drugs are available, their efficacies are limited by the existence of drug-resistant parasites worldwide (including Brazil), especially in the case of *Plasmodium falciparum* [2, 3]. In this context, it is needed either the discovery of new drugs associated with new targets or to improve determined anti-malarial drug class.

The antiparasitic activity of hydroxy-naphthoquinones derivatives is already known, with studies confirming its efficacy against *Leishmania braziliensis* and *Leishmania amazonensis* [4], *Trypanosoma cruzi* [5] and *P. falciparum* [6–10]. Its mechanism of action was already proposed by means of the inhibition of the mitochondrial electron carrier chain [11].

The biosynthesis of isoprenoids pathway in *P. falciparum* are excellent therapeutic targets because they are different or even absent in the human host. Their many functions are quite important for the parasite’s survival [12]. Vitamins E and K, belonging to the family of isoprenoid, has an isoprenic chainsaw from MEP pathway. These vitamins are essential components for the cellular machinery found in all organisms. The menaquinone (MQ) (vitamin K_2_) is employed as electron carriers required for the mitochondrial respiratory chain [13–15], with the α-tocopherol (TC) representing more bioactivity of vitamin E, protecting the membranes against peroxidation [16].

According to the Resolution CNS 251/97, pre-clinical research is the first step in the study on the development of new drugs. This approach should provide information for possible therapeutic application, besides to predict some risks such as toxicity and adverse effects [17]. The absorption, distribution, metabolism, and elimination (ADME) characteristics of a drug are conventionally viewed as an important part of the drug development [18]. Unfavourable ADME is the leading cause of costly and clinical failures in the development of new chemical products during the drug development projects [19–21]. It is believed that around 40–50% of study in clinical phases fail due to toxicity and pharmacokinetic difficulties [18, 22]. Therefore, there is an increasing interest in the early ADME prediction of drug candidates. Besides of drug-likeness and ADME data predictions, it has been demonstrated that the anti-malarial activity and interference with the synthesis of isoprenic precursors of three derivatives of hydroxy-naphthoquinones against *P. falciparum*.

## Methods

### Compounds

The tested hydroxy-naphthoquinone derivatives were setting in the School of Pharmaceutical Sciences of Ribeirão Preto, University of São Paulo, Brazil [9], namely: 2-aniline-3-hydroxy-1,4-naphthoquinone, 2-chloro-aniline-3-hydroxy-1,4-naphthoquinone, 4-methoxy-aniline-3-hydroxy-1,4-naphthoquinone and 2.6-dimethyl-aniline-3-hydroxy-1.4-naphthoquinone, from now on designated as **4a**–**4d**, respectively. Compounds **4a** and **4c** were selected for all tests since they are soluble in the solution with a maximum of 1% DMSO, presenting satisfactory results in the screening tests.

### Drug-likeness and ADME predictions

In drug discovery projects, the fastest efficient method to guide the evaluation of the drug-like properties of compound libraries is to apply simple rules that compare their physicochemical properties with those presented by existing drugs with suitable pharmacokinetic profile [23]. To do that, we applied the Lipinski’s rule of five (R05), Lead-like soft, Drug-like soft, REOS and ZINC rules in compounds **4a** and **4c** using FAF-Drugs3 web-server [24]. It was also used the MarvinSketch Version 5.0 (http://www.chemaxon.com) [25] and ALOGPS 2.1 [26] software programs to determine the following descriptors (structural and physicochemical properties): molecular weight (MW), numbers of hydrogen bond donors (HBD), hydrogen bond acceptors (HBA), number of rotatable and rigid bonds, flexibility, number of carbon/hetero-atoms, value of ratio Het/c (H/C ratio), number/maximum size of rings, number of atoms with a net charge, sum of formal charges, carbon bond saturation (Fsp3), number of STEREOCENTRES, molecular polarizability, molar refractivity, polar surface area (PSA), octanol/water partition coefficient (logP), octanol–water distribution coefficient (logD) and aqueous solubility (logSw). Afterwards, an oral absorption/bioavailability evaluation considering Lipinski was performed, Veber, Egan, and Bayer (Bayer Oral Physchem Score) rules. Finally, we run ADME by PreADME program [27].

### *Plasmodium falciparum* cultures

Experiments were performed using chloroquine-sensitive 3D7 and chloroquine-resistant Dd2 strains. Parasites were cultured using the method of Trager and Jensen, with previously described modifications [28]. Synchronized ring phase cultures were obtained by two consecutive treatments at intervals of 48 h with a 5% (m/v) solution of D-sorbitol (Sigma-Aldrich) [29]. All tests described below were performed in triplicate and repeated at least three times.

### Antiplasmodial activity

Drugs were diluted with 0.1% DMSO, followed by a serial 1:2 dilution (nine concentrations) ranging from 160 to 0.6 µM in the complete culture medium. Anti-malarial activity was performed using SYBR Green I assay [30] in 96-well culture plate where the compounds were added to the culture of *P. falciparum* with predominantly rings of 1% parasitaemia and 3% haematocrit. The microplate was incubated at 37 °C for 48 h under an atmosphere having a low oxygen level (5% CO_2_). Chloroquine was used as a control, in standard concentrations. After 48 h of incubation, cells infected with 3D7 and Dd2 strains were analysed using the SYBR Green I. Values of IC_50_ were calculated using GraphPad PRISM software. Compounds were ranked, according to its activity, as being high activity (IC_50_ ≤ 10 µg/ml); moderate activity (10 < IC_50_ < 100 µg/ml); and low activity (IC_50_ > 100 µg/ml) [31].

### Treatment com hydroxy-naphthoquinones and metabolic labelling

Synchronous cultures of *P. falciparum* with 10% parasitaemia of schizonts were treated with 6.5 µM and 3.75 µM, for **4a** and **4c**, respectively, corresponding to 80% of IC_50_. A flask was kept untreated and is considered the positive control. After 36 h, the culture was labeled with [1-(*n*)-^3^H]GGPP (3.125 µCi/ml) in normal RPMI 1640 medium [32] during the last 16 h. The schizonts were purified by magnetic column (MACS Separation Columns “CS”, Miltenyi Biotec) [33]. The parasitaemia was estimated by microscopic examination of Giemsa-stained smears and the volume was adjusted of purified culture, according to the control, so that equal numbers of treated and untreated parasites were applied. After metabolic labelling and purification, lyophilized schizonts were used for extraction of MQ and TC. The final extract was injected into the HPLC-RT [34].

### Reversed-phase high-performance liquid chromatography (RP-HPLC)

The molecules of MQ and TC were purified by an isocratic system with mobile phase methanol: ethanol (50:50 v/v), flow 0.5 ml/min and detection performed by UV absorption spectrum at a wavelength equal to 270 nm. The mobile phase was filtered on a PTFE (solvent A) and NYLON (solvent B) membrane of 0.20 µM. Radioactive fractions were dried by evaporation at 24 °C and resuspended in scintillation fluid. Quantification of the radioactivity (c.p.m.) was performed on the device Beckman 5000 β-radiation scintillation counter (Beckman, CA, USA) [34].

### Statistical analyses

The IC_50_ was estimated by linear interpolation as compared to the drug-free controls, using the software GraphPad PRISM software. Comparative statistical analysis of the peak areas from HPLC chromatograms of samples treated with compounds **4a** and **4c** versus untreated samples were performed for ANOVA, (95% confidence interval), using the statistical software program Assistat 7.7 beta [35]. The results were considered statistically significant at P-value ≤ 0.05.

## Results

**Fig. 1:**
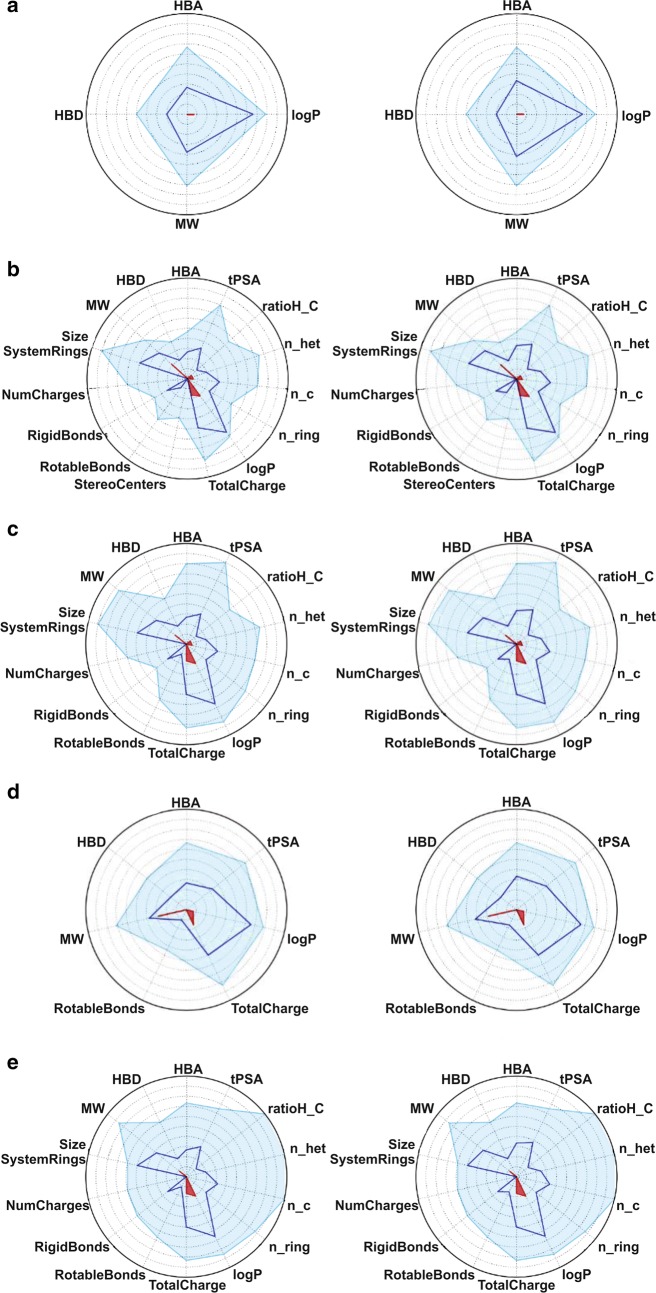
Radar plots positioning compounds values within the selected filter ranges (pale blue and red). The blue line of compound values should fall within the **a** Lipinski RO5, **b** Lead-like soft, **c** Drug-like soft, **d** REOS and **e** ZINC filter area bounded by the light blue line

**Fig. 2:**
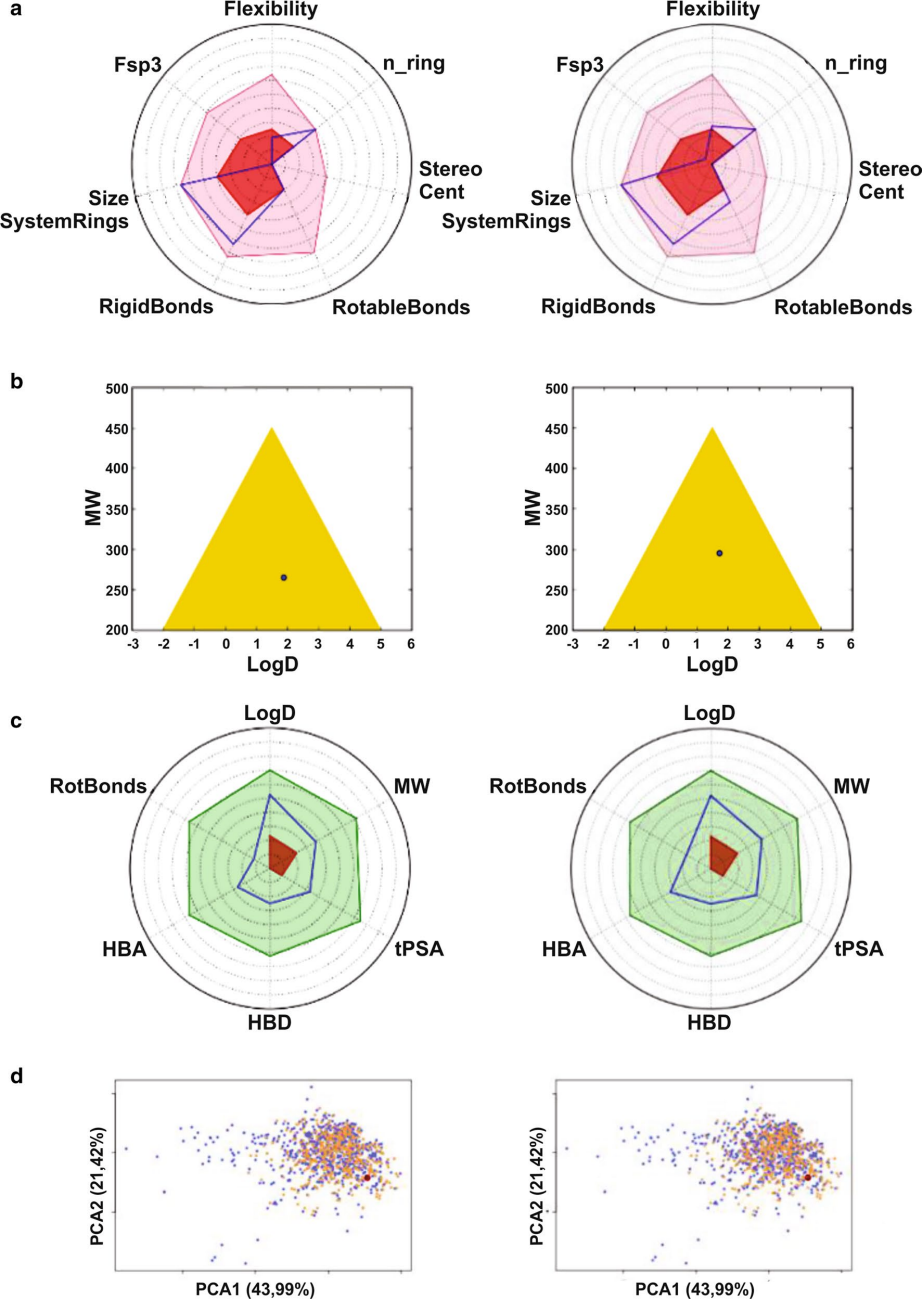
Graphical reports of compounds **4a** and **4c**. **a** Compound values (blue line) superimposed on an oral library with min and max ranges (pink and red) during a compound complexity analysis. **b** Golden Triangle Rule evaluation: compounds located in the yellow triangle are likely to have an optimal permeability and a good metabolic stability. **c** For an oral absorption evaluation, compound values (blue line) should fall within RO5 and Veber rules area (light green line). **d** The Oral Property Space, obtained by applying a Principal Component Analysis of the 15 principal physicochemical descriptors of user’s compound (red), compared to two oral sub-libraries extracted from eDrugs (blue) and DrugBank (orange)

All physicochemical descriptors and ADME predictions calculated for compound **4a** and **4c** are shown in Table 1. Figure 1 depicts the PhysChem filters positioning of candidates in (a) Lipinski RO5 [36], (b) Lead-like soft, (c) Drug-like soft, (d) REOS and (e) ZINC rules designed by combining several drugs’ physicochemical parameters. The R05 and Veber rules area obtained with the following descriptors ranges: logP (− 2 to 5), molecular weight (150 to 500), tPSA (20 to 150), rotatable bonds (0 to 10), H-bonds acceptors (0 to 10) and donors (0 to 5). The complexity, permeability/metabolic stability (Golden Triangle Rule), oral absorption estimation and the oral bioavailability evaluation of compounds are showed in Fig. 2.

**Table 1:** Physicochemical descriptors and ADME-related properties of compounds **4a** and **4c**

Descriptors	Compounds	
	**4a**	**4c**
MW	265.26	295.29
HBD	2	2
HBA	4	5
Rotatable bonds	2	3
Rigid bonds	19	19
Flexibility	0.10	0.14
Carbon atoms	16	17
Hetero atoms	4	5
H/C ratio	0.25	0.29
Ring	2	2
Max size ring	10	10
Total charge	0	0
Fsp3	0.00	0.06
Stereo centres	0	0
Polarizability (cm^2^/V)	28.17	30.70
Refractivity (m^3^/mol)	78.11	84.57
PSA (Å^2^)	66.4	75.63
logP	3.24	3.21
logD	1.88	1.72
logSw	− 3.68	− 3.75
HIA (%)	93.67	93.85
Caco2 (nm/s)	20.20	21.55
MDCK (nm/s)	343.27	208.47
logKp	− 3.59	− 3.60
BBB (C. brain/C. blood)	0.63	0.07
PPB (%)	86.91	85.73

**Fig. 3:**
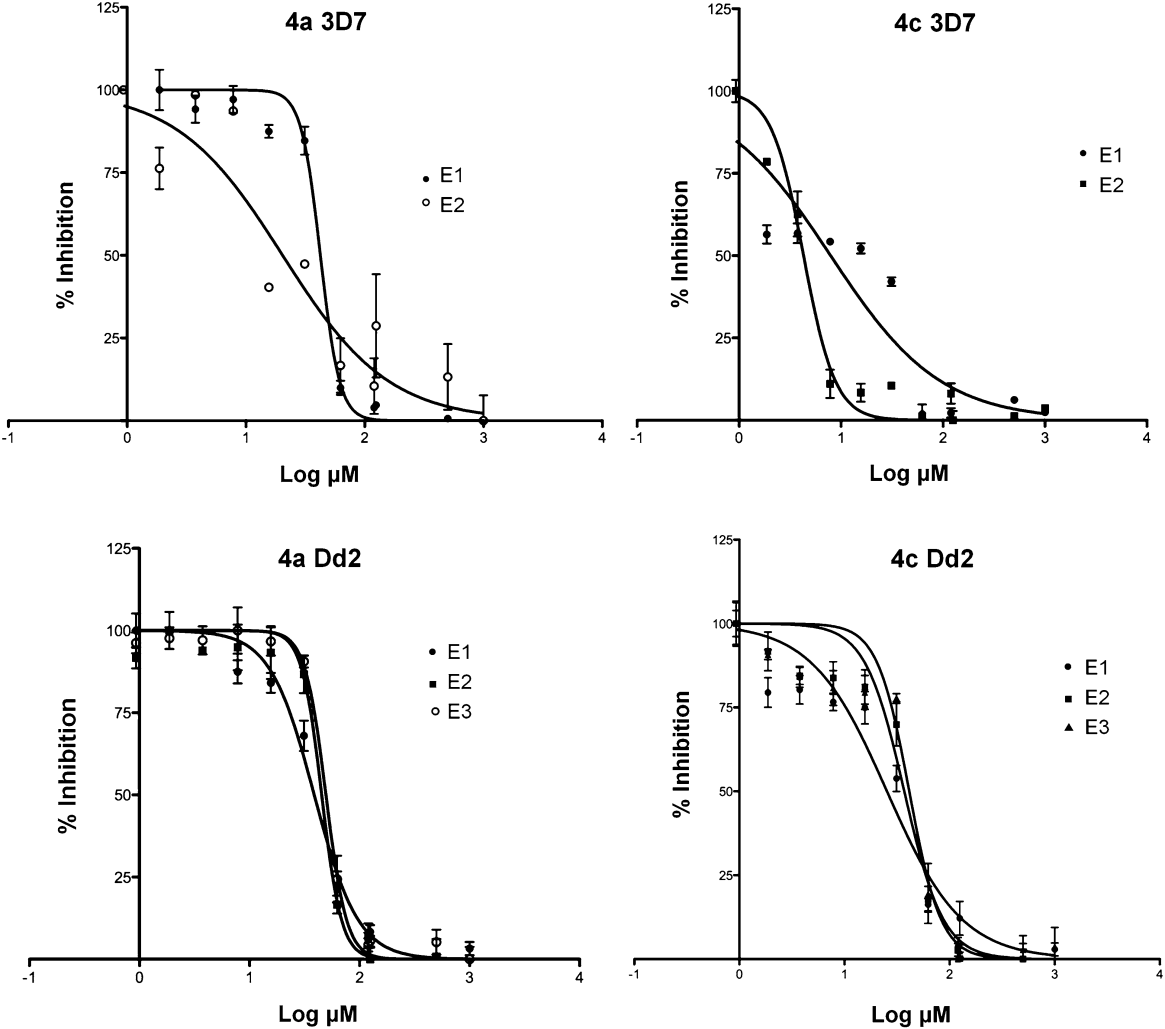
Dose–response curve of **4a** and **4c** compounds on *P. falciparum* 3D7 and Dd2 strains infected erythrocytes. Values are mean ± SEM in representative experiment in triplicate. E1, E2 and E3 are repeats of the tests

The IC_50_ for **4a** and **4c** were determined by in vitro growth inhibition assay using *P. falciparum* 3D7 and Dd2 strains. Table 2 and Fig. 3 present the mean IC50 values for **4a**, **4c**, and chloroquine (reference anti-malarial drug).

**Table 2:** The IC_50_ mean for **4a** and **4c** compounds and chloroquine

*P. falciparum*	**4a**		**4c**		Chloroquine	
	Mean IC_50_ (μg/ml)	Variance	Mean IC_50_ (μg/ml)	Variance	Mean IC_50_ (μg/ml)	Variance
3D7 strain	2.99^a^	2.41–3.57	2.06^a^	1.47–2.65	0.012^a^	0.007–0.017
Dd2 strain	11.79^b^	10.36–13.22	10.10^b^	7.72–12.48	0.15^a^	0.12–0.18

IC_50_ (inhibitory concentration 50%); high activity^a^ (IC_50_ ≤ 10 μg/ml); moderate activity^b^ (10 < IC_50_ < 100 μg/ml) (Meneguetti et al. [31])

**Fig. 4:**
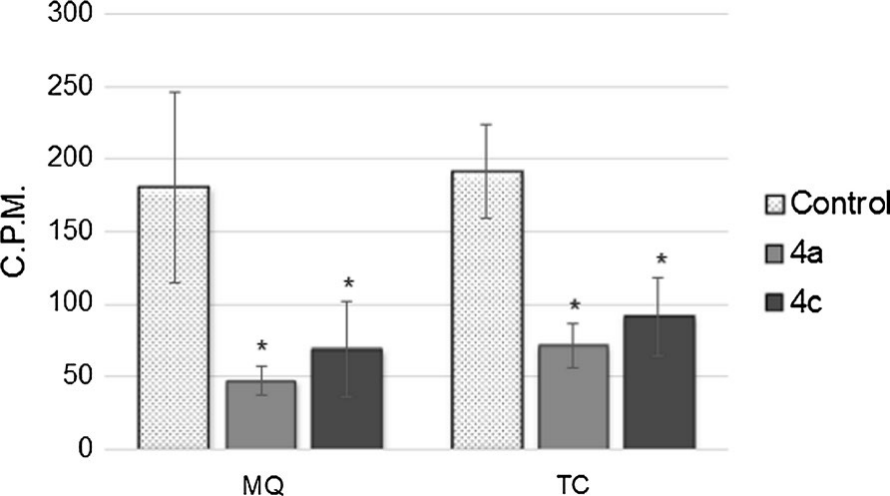
Menaquinone and tocopherol level change in response to **4a** and **4c** compounds treatment. Comparison between treatment with 6.5 µM of **4a** and 3.75 µM of **4c** and untreated (control) culture, after purification by RP-HPLC. The analyzed fractions, corresponding to the retention times of the standards are: MQ—16 min and TC—17 min. An asterisk indicates a significant difference compared to untreated controls (*P* values of < 0.05). *MQ* menaquinone, *TC* tocopherol

Change in MQ and TC biosynthesis resulting from treatment with the hydroxy-naphthoquinone derivatives is shown in Fig. 4. The activity (in c.p.m.) of the fractions corresponding to the retention times of MQ 15 and TC 16 were compared with those of the corresponding fractions from untreated controls. Biosynthesis of MQ and TC were inhibited in 71.2% and 62.7% (58.1% and 49.6%) by derivative **4a (4c)**.

## Discussion

Due to the global spread of the multidrug-resistant *P. falciparum* to usual anti-malarials, the disease control has been hampered mainly in African and Asian countries, by means of the development of new anti-malarial drugs and the search for new therapeutic targets. However, for a new drug to be released to the custom market, numerous studies are required to prove its safety and efficacy. The first step is the pre-clinical in silico, in vitro and in vivo tests, depicting the relevance of the findings, the possible therapeutic applications as well as previewing some risks with its use.

The physicochemical property filters are used in drug discovery and drug development to narrow down the scope of molecules [37, 38]. They estimate the drug-likeness profile (Druglikeness prediction) of the active compounds by considering their physical and chemical properties [39–41]. After analysing the physicochemical descriptors values calculated, it was observed that the components **4a** and **4c** meet the criteria for the Lipinski’s R05, Lead-like soft, Drug-like soft, REOS and ZINC rules [42] (see Table 1 and Fig. 1).

Figure 2 shows that both compounds reach the criteria for the number of system ring, stereocentres, rotatable and rigid bonds, as well as the maximum size of the system rings (number of system ring, stereocentres, rotatable and rigid bonds, the flexibility and the maximum size of system rings) successfully [43]. In this study, compound values are superimposed on a database comprising currently 1790 chemical structures of drugs [44]. By Golden Triangle Rule, both compounds have an optimal permeability (low clearance) and a good metabolic stability. They are believed to be the designed drug from the most ligand and lipophilic efficient lead into the centre of the Golden Triangle and should provide the maximum potency, stability, and permeability [45].

To further estimate the druggability of these naphthoquinones derivatives were report their ADME descriptors and profiles and potential biological activities using an in silico approach are depicted in Table 1. While the likelihood of drug absorption has been described in compliance to the Ro5, Veber rule, human intestinal absorption (HIA), skin permeability (logKp), colorectal carcinoma (Caco2) and Madin-Darby canine kidney (MDCK) cells permeability, the drug distribution was modeled using the blood/brain partition coefficient (BBB) and the coefficient of binding to human serum albumin (PPB).

Human skin has a low permeability for most of the foreign substances, which are unable to penetrate and diffuse through the skin. Pre ADME predicts in vitro skin permeability and the result value is given as logKp (permeability coefficient). In this work, the compounds **4a** and **4c** show logkp de − 3.59 e − 3.69 respectively, delimited within the scale of − 8 to − 1, common drug 95% [46]. As a comparative test, the water, for example, has predicted ranging from logkp − 7.39 to − 6.27 [47].

The oral absorption evaluation is one of the most influential ADME characteristics in the early stages of lead discovery and optimization [48]. The compounds **4a** and **4c** are well absorbed in the human intestine, with values for HIA being 93.67% and 93.85% respectively. The permeability coefficient in human intestinal epithelial (Caco-2) and renal (MDCK) cell presented intermediate values for **4a** (**4c)**, lying within the recommended ranges of 95% for known drugs [49–51].

The RO5 and Veber rules comprise a set of rules that attempt to predict if a molecule could be administered orally [52]. As can be seen in Fig. 1, compounds **4a** and **4c** fail within R05 and Veber rules area obtained with the analyzed descriptors. In addition, the compounds examined successfully meet the proposed criteria for Egan et al. [53] and Lobell et al. [54], which states that for a compound be transcellularly absorbed from the gastrointestinal tract into systemic circulation it must be reasonably soluble in aqueous solution, although should not be too polar (PSA), too lipophilic (CLOGP), too large (MW) or too flexible (rotatable bonds) to pass cellular membranes.

A good oral bioavailability reduces the amount of an administered drug necessary to achieve a desired pharmacological effect and, therefore, could reduce the risk of side-effects and toxicity. A compound positioning within 466 and 916 orally bioavailable compounds extracted from the DrugBank and e-Drug3D databases respectively [43, 55] are presented in a graph obtained by applying the PCA (Principal Component Analysis) of the 15 main physicochemical descriptors of these molecules.

The plasma protein binding (PPB) and blood–brain barrier (BBB) penetration [56, 57] were calculated for detecting its distribution into the human body. The PPB values obtained for **4a** and **4c** indicate that the chemicals are moderately bound to albumin. An analysis of the PPB’s percentage distribution among some therapeutic drugs showed that chemotherapeutics presenting PPB > 90% binding can be classified as therapeutic drugs [58]. Further, a BBB penetration value determined in this work indicates a middle (low) absorption to central nervous system-CNS by studied molecules [59]. This is crucial in the pharmaceutical sphere because CNS-active compounds must pass across it, while CNS-inactive compounds do not, in order to avoid any CNS side effects.

According to the System Biopharmaceutics Classification (BCS), which ranks drugs based on their aqueous solubility and intestinal permeability through the correlation between in vitro dissolution and bioavailability of the drug in vivo [60, 61], atovaquone is classified in class II, which includes drugs with low solubility, having high permeability and good absorption [62].

Atovaquone, as well as the tested compounds, is a 3-substituted-2-hydroxy-1.4-naphthoquinone complex. It presents an excellent anti-malarial activity but has poor pharmacological properties, such as low bioavailability and high binding to plasma proteins due to its lipid solubility [63]. To improve its bioavailability, some analogs of atovaquone were created whose changes were made in the naftoquinoidal group, especially the alkyl side chain. It is known that modification of this chain can alter the activity of the drug [64]. The 4a and 4c compounds tested in vivo [9] presented interesting antiplasmodial activity, using the murine *Plasmodium berghei* model. The output of these experiments may result in low bioavailability and/or high plasma protein binding, and atovaquone. The low bioavailability of atovaquone, and probably of our compounds, is due to the limited solubility in aqueous media, although conferring good intestinal permeability. Tests that include solubility enhancement techniques through physical changes, chemical or by various methods has been not only already studied, but also be employed, since approximately 70% of new drug candidates and more than 40% of the newly developed chemical formulas in the pharmaceutical industry are practically insoluble in water [65, 66].

The antiplasmodial in vitro activity of derivatives hydroxy-naphthoquinones was satisfactory against the 3D7 strain (sensitive chloroquine), being classified as high activity anti-malarial compounds. For the Dd2 strain (multidrug resistant), the IC_50_ values were fivefold higher; being classified as moderately active against this strain.

In a study which tested over 10 modified molecules from hydroxy-naphthoquinones, the IC_50_ values were greater than 50.0 μM [8]. Another study tested five derivatives hydroxy-naphthoquinone with radical aquil, and only in one of the derivatives, whose radical was a phenyl grouping, was very active in the nanomolar order of magnitude [67]. Derivatives of 1.4-naphthoquinones, which was a radical trifluormethylbenzene, showed IC_50_ in the nanomolar order of magnitude. This high anti-malarial activity is probably due to the instability of the radical trifluormethylbenzene, which leads to the formation of free radicals that are toxic to the parasite [11]. However, a study with three derivatives hydroxy-naphthoquinone aminated, showed that only one was effective against the *P. falciparum* NF54; strain sensitive to most anti-malarials [7]. Their radical was a phenylpiperazine on carbon 6, with the same root added to other lead compounds. This demonstrates antiplasmodial effectiveness in these other changes, confirming a relationship with this radical activity. The phenylpiperazine is attached to a piperazine phenylaniline, showing similarity with radicals used in structural changes, as in the present study, which is aniline derivatives and associated with the effectiveness of that radical. In another study, eight derivatives of 2-hydroxy-3-methylamino-1.4-naphthoquinone were tested, and five were active, exhibiting IC_50_ values less than 30 μM against *P. falciparum* W2 (chloroquine-resistant strain) [10].

Pyrimethamine, an anti-malarial already used in combination with sulfadoxine, has a higher IC_50_ when compared to present work and narrow therapeutic window [68], confirming the importance of obtained results. Several studies have shown that the structural changes in the molecule have reduced its cytotoxic effect while improving either the biological activity [69, 70] or the pharmacokinetic and pharmacodynamic properties [69].

Concerning the likely target of the derivatives of hydroxy-naphthoquinones tested, the results obtained in this study suggest the biosynthesis of isoprenoids as a possible target, since the two products of this pathway had a significantly decreased production: the vitamin K_2_ (MQ) that act as an important electron receiver of the respiratory chain [34] and the α-tocopherol that protects membranes from lipid peroxidation [32]. The α-tocopherol also acts as an antioxidant and free-radical scavenger, can efficiently quench O_2_ and scavenge various radicals released during oxidative stress [71]. A candidate for an anti-malarial drug that acts simultaneously in the synthesis of these two molecules is interesting because it can trick the parasite resistance mechanism, both interfering in the cellular respiration when in plasma membrane integrity. Furthermore, due to its absence in humans, the MEP pathway can be considered as an important drug target for malaria, although additional studies should be performed to confirm which enzyme is inhibited, after the addition of isopentenyl-PP [12] or geranylgeranyl pyrophosphate synthase (GGPP) to the cultures [72]. Due to their structural similarity with atovaquone, these novel compounds may probably act through a similar mechanism. Atovaquone is believed to inhibit electron flow in aerobic respiration, by binding to cytochrome b in place of ubiquinone in the parasite mitochondria. Such inhibition hampers the activity of dihydroorotate dehydrogenase, an enzyme linked to the mitochondrial electron transport system that is required in the de novo synthesis of pyrimidines [73]. Additionally, the redox potential of hydroxy-naphthoquinones is suggested to cause an oxidative stress which may also be responsible for some antiparasitic activity of this class of compounds. However, one recent study found that 3-[4-(trifluoromethyl)benzyl]-menadione do not inhibit the mitochondrial electron transport chain, but exert their anti-malarial activity as redox-active subversive substrates [11].

The compound **4c** presents better in vitro results, since it has the lowest IC_50_ and interfered in the biosynthesis of the two products of the isoprenoid pathway. In this sense, it provides a better activity of the compound, preventing escape mechanisms by the host avoiding the oxidative stress process, despite as much as tocopherol acts as an antioxidant. In silico computations have been carried out for the calculation of the physicochemical and subsequent drug-likeness and ADME predictions. Satisfying a number of rules and physicochemical filter (see “Methods”), the compounds present acceptable drug-like profile, specifically structural alerts, good oral bioavailability, permeability, and distribution. Modifications in the structure of compound **4c** as the salt formation and complexation with β-cyclodextrin can be interesting because solubility enhancing techniques can promote the efficacy of the compound leading to an increase in bioavailability as a consequence of improved solubility.

## Conclusions

Thus, the molecules tested in the present study, although presenting lower anti-malarial activity than some of the usual class of anti-malarials, could be used as prototypes for future programmes that could develop safe and effective drugs using their pharmacophores with those of other drugs into synergistic conjugates.
